# Factors Associated With Self‐Medication to Mitigate Vaccine Reactions After COVID‐19 Vaccination: A Prospective Cohort Study

**DOI:** 10.1002/pds.70372

**Published:** 2026-04-10

**Authors:** Maximilian Blattner, Ingmar Schäfer, Yvonne Nestoriuc, Christian Büchel, Martin Scherer, Jan Hendrik Oltrogge‐Abiry

**Affiliations:** ^1^ Department of General Practice and Primary Care University Medical Center Hamburg‐Eppendorf Hamburg Germany; ^2^ Clinical Psychology and Psychotherapy Helmut‐Schmidt‐University/University of the Federal Armed Forces Hamburg Hamburg Germany; ^3^ Institute of Systems Neuroscience University Medical Center Hamburg‐Eppendorf Hamburg Germany

**Keywords:** nonprescription drugs, patient reported outcome measures, primary health care, prospective cohort studies, quality in health care, self medication

## Abstract

**Objective:**

Management of vaccine reactions with analgesics/antipyretics can enhance acceptance and adherence to future vaccinations. However, inappropriate self‐medication may pose health risks, particularly for individuals with chronic conditions. The aim of this study was to identify demographic, clinical, and psychosocial factors associated with self‐medication behavior after COVID‐19 vaccinations.

**Methods:**

Monocentric prospective cohort study at a vaccination center in Germany between August 16 and 28, 2021 with adults receiving a second dose of mRNA‐based COVID‐19 vaccines. We used linear regression analyses to examine the association of self‐reported factors with the amount of self‐medication after vaccination.

**Results:**

A total of 1616 participants (median age 34 years, 52% females) were included, with 536 (33.2%) reporting the use of self‐medication. Used substances were nonsteroidal anti‐inflammatory drugs *n* = 345 (21.3%), paracetamol *n* = 186 (11.5%), acetylsalicylic acid *n* = 48 (3.0%), and metamizole *n* = 18 (1.1%). The highest intake of any analgesic/antipyretic was observed on the first day after vaccination *n* = 393 (24.3%). Factors associated with increased self‐medication included premedication with analgesics/antipyretics (*B*: 0.545; 95% CI: 0.442; 0.651), administration of the mRNA‐1273‐vaccine compared to the BNT162b2‐vaccine (*B*: 0.293; 95% CI: 0.188; 0.397), female gender (*B*: 0.159; 95% CI: 0.07; 0.249), prior experiences of vaccine reactions (*B*:0.117; 95% CI: 0.054; 0.18), expectations of vaccine reactions (*B*: 0.063; 95% CI: 0.003; 0.123), and the presence of common chronic conditions. A higher satisfaction with the vaccination process (*B*: −0.129; 95% CI: −0.222; −0.039) was associated with lower rates of self‐medication.

**Conclusions:**

Factors influencing self‐medication behavior after vaccination exist and could be assessed prior to vaccine administration. The observed association of previous experiences as well as expectations of vaccine reactions suggests that nocebo effects contribute to self‐medication practices.

## Key Points

- The study evaluated various demographic, clinical, and psychosocial factors associated with self‐medication after COVID‐19 vaccination.
- Female gender, the mRNA‐1273 vaccine type, premedication with analgesics/antipyretics as well as chronic conditions (back pain and depression) were associated with more self‐medication.
- Psychosocial factors, including negative experiences and expectations, increased the likelihood of self‐medication, indicating the role of nocebo effects.
- Higher satisfaction with the vaccination process reduced self‐medication indicating the value of procedural context factors of the vaccination process.
- Awareness of these factors could inform safer and more individualized self‐medication guidance for patients, especially those with chronic conditions.

## Plain Language Summary

This study investigated why people use painkillers to manage vaccine reactions after their vaccination. We surveyed 1616 adults in Germany who received their second COVID‐19 mRNA vaccine shot. About one‐third of participants reported using painkillers like ibuprofen or paracetamol, especially on the first day after vaccination. People were more likely to use these drugs if they had already used them previously, were women, had stronger vaccine reactions at their first vaccination, expected more vaccine reactions, or received the Moderna vaccine instead of Pfizer/Biontech. Chronic health problems, like back pain or depression, also increased the chances of self‐medication. Those satisfied with the vaccination process were less likely to self‐medicate. Recognizing these factors could help healthcare providers assess their patients before planned vaccination and provide tailored advice on self‐medication to manage vaccine reactions.

## 1 Introduction

Vaccines are regarded as an effective public health instrument with a proven effectiveness in controlling outbreaks of infective diseases, but their success depends on public acceptance [1]. Concerns about vaccine reactions have already been identified as a source of vaccine hesitancy in various contexts [2].

During the COVID‐19 pandemic acceptance of vaccines in industrialized countries fell short of the set targets, despite a sufficient availability [3]. Concerns about potential vaccine side effects were identified as common reasons for vaccine hesitancy [4]. Side effects following a COVID‐19 vaccination include systemic reactions such as cephalalgia, myalgia, chills, and fever as well as local reactions at the injection site [5, 6]. Notably, vaccine types differed in reactogenicity; for instance, the mRNA‐1273 vaccine showed a higher reactogenicity compared to BNT162b2 [7, 8].

Various clinical guidelines mention the use of analgesics/antipyretics as therapeutics to manage vaccine reactions if needed to manage vaccine reactions but recommend against their prophylactic use [9, 10]. Of note, it was hypothesized that analgesics/antipyretics could attenuate antibody responses [10, 11, 12]. However, a recent review concluded that there is not enough evidence to assume a clinically meaningful impact of analgesics/antipyretics on vaccine efficacy [13]. Consequently, some authors have even proposed that promoting analgesics/antipyretics to reduce vaccine reactions could enhance vaccine acceptance as part of pandemic preparedness strategies [14].

On the other hand, the widespread availability and promotion of analgesics/antipyretics may cause harm due to misuse, abuse, and dependence [15]. For patients with chronic conditions there may be a need for individual guidance on drug intake by their primary care physicians in terms of dose recommendations, possible drug interactions or even contraindications due to co‐morbidities. For instance, paracetamol is contraindicated in advanced liver disease, and the increased risks of gastric ulcers and renal failure with nonsteroidal anti‐inflammatory drugs (NSAIDs) in patients with multimorbidity [16, 17].

Therefore, it would be valuable for primary care physicians to be able to assess a patient's likelihood of self‐medication based on anamnestic factors that could be gathered prior to a vaccination.

However, there is no international consensus on the definition of self‐medication and presumed predictors vary strongly between developed and developing countries and their respective health care systems [18, 19]. Recurring factors that were associated with self‐medication of analgesics/antipyretics in reports from European countries were gender, age, educational level, health literacy, and chronic conditions [20, 21, 22].

In addition, evidence is indicating that the self‐reported vaccine reactions (that would be targets of analgesics/antipyretics usage) are to some extent based on nocebo effects [23]. Nocebo effects emerge through three main mechanisms: learning from prior negative experiences, expectations and misattribution of pre‐existing symptoms [24, 25, 26, 27]. In an already published analysis, we showed that expectations and pre‐experiences can influence the severity of vaccine reactions [28].

Therefore, we hypothesized that there are not only demographic and clinical factors but also known psychosocial factors of nocebo effects that would influence self‐medication after a vaccination. For the present analysis, we assumed that such factors would likewise show an association with the amount of self‐medication with analgesics/antipyretics after a vaccination. Consequently, our study aim was to identify associations between these factors and reported self‐medication.

## 2 Methods

### 2.1 Study Design, Setting, and Participants

We conducted a longitudinal cohort study at Germany's largest COVID‐19 vaccination center in the federal state of Hamburg [29]. The center operated from January 5 to August 31, 2021, daily from 8 00 a.m. to 8:00 p.m. with up to 7000 vaccinations per day. Participants were recruited from 16 to August 28, 2021. All individuals aged 18 or older with the capacity to consent and sufficient German language skills were eligible for the study if they received a second dose of *BNT162b2* (Pfizer‐BioNTech) or *mRNA‐1273* (Moderna). During the study period, most visitors were receiving their second dose of an mRNA vaccine (76.6% of all visitors). An additional description of the setting and recruitment process is published elsewhere [28]. Briefly, a study leaflet including a QR code for the installation of the smartphone application m‐Path (UK Leuven) was given to all participants at the vaccination center. Participants were asked for informed consent within the app before they filled out the baseline questionnaire during a 15‐min waiting time immediately after vaccination. After completion of the baseline questionnaire the application activated a time schedule for follow‐up surveys for the consecutive 7 days beginning at 9:00 p.m. on the day of recruitment (Day 1) and at 6:00 p.m. on Day 2–7. Participants were reminded via push messages to answer the follow‐up surveys. The study was approved by the local Psychological Ethics Committee at the Center for Psychosocial Medicine of the University Medical Center Hamburg‐Eppendorf on April 28, 2021 (LPEK‐0312).

### 2.2 Dependent and Independent Variables

The dependent variable was defined as the self‐reported number of days under self‐medication with analgesics/antipyretics to mitigate vaccine reactions (range: 0–7 days).

Independent variables were grouped into sociodemographic, clinical, and psychosocial factors. Demographic variables included age (continuous), gender (reference: female), living arrangements (reference: living with others), educational level (reference: low education), and migration status (reference: nonmigrant). Educational level was assessed by the Comparative Analysis of Social Mobility in Industrial Nations classification (CASMIN) [30] and the migration status by country of birth of study participants and their parents.

Clinical factors at baseline were self‐reported problems (based on a list of 13 medically treated health problems) [31], reported premedication with analgesics/antipyretics two weeks prior to vaccination (reference: no premedication) and the administered vaccine type mRNA‐1273 (reference: BNT162b2).

Psychosocial factors included the experienced severity of vaccine reactions at first vaccination and the participants´ expectations regarding benefits as well as their risk for vaccine reactions of the second vaccination. We used 11‐point Likert scales ranging from “no complaints” to “maximum discomfort,” “no benefit” to “maximum benefit” and “no risk” to “maximum risk,” respectively. We screened for existing anxiety and depression with the four‐item version of the Patient Health Questionnaire [32] and the Somatosensory Amplification Scale for the participants´ tendency to detect somatic sensations and experience them as unusually intense [33]. Finally, we asked for the participants´ satisfaction with the overall process of the vaccination at the vaccination center (11‐point Likert scale; “not satisfied” to “very satisfied”).

For the full questionnaire, see Table S3.

### 2.3 Statistical Analysis

We used descriptive analyses to show the distribution of categorical variables, mean (SD) for normally distributed continuous variables and median (IQR) for non‐normally distributed variables. The unadjusted association of each factor with the duration of self‐medication was determined by linear regression analyses. We used a multiple linear regression analysis to determine adjusted associations of our self‐reported factors with the days under self‐medication after vaccination. The selection of variables for the final model was performed using forward inclusion: variables were added stepwise if they met a significance threshold of *p* < 0.05. A model with all variables included was tested for collinearity using tolerance (< 0.1) and variance inflation factor (> 5) and no indication of multicollinearity was found except for the CASMIN variables what we interpreted as structural multicollinearity due to dummy coding (Table S1). As a sensitivity analysis, we conducted a binary logistic regression with self‐medication dichotomized (no medication vs. ≥ 1 day) to assess the robustness of findings (Table S2). The results are reported as regression coefficients (*B*) with corresponding 95% confidence intervals (CIs). All data were analyzed using SPSS 28 (IBM, New York, USA).

## 3 Results

### 3.1 Recruitment Process

**FIGURE 1:**
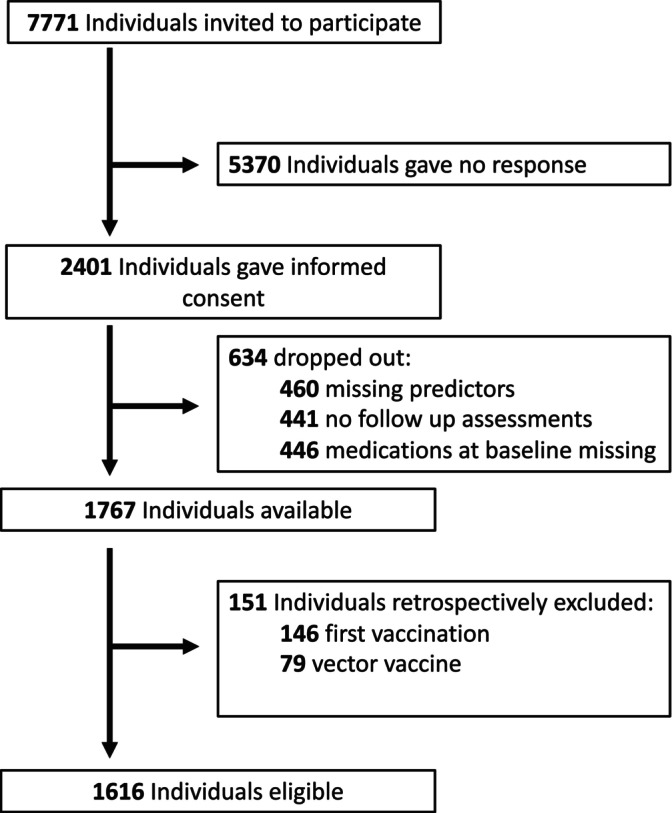
Recruitment of study participants.

The recruitment process is depicted in Figure 1. In the study period, 7771 individuals at the vaccination center were invited to participate. Of these, 2401 consented to participate in the study and activated the study app in their smartphones. Of these, 634 provided incomplete information and had to be excluded afterwards. In addition, 151 individuals were excluded because they either received their first vaccination or a vector vaccine. Consequently, 1616 participants (20.8% of all vaccination center visitors) could be included in the analysis.

### 3.2 Study Population

A depiction of the study population is shown in Table 1. The median age was 34 (IQR, 27–44) years; 840 (52.0%) were female, and 776 (48.0%) were male. More than half of the sample (894, 55.3%) reported having a tertiary level education, and (647, 40.0%) had completed secondary level schooling, and (75, 4.6%) reported primary education.

**TABLE 1:** Patient characteristics.

Characteristic	Values
Total no. of patients	1616
Age, median (IQR) (years)	34 (27–44)
Gender	
Female	840 (52)
Male	776 (48)
Living arrangements	
Living together with others	1175 (72.7)
Living alone	441 (27.3)
Educational level (pursuant to CASMIN)	
Higher or lower tertiary education	894 (55.3)
Secondary school certificate or A‐Level equivalent	647 (40.0)
Inadequately completed, general elementary or basic vocational	75 (4.6)
Migration status	
Study participant and both parents born in Germany	1163 (72.0)
Study participant born in Germany and at least 1 parent born abroad	229 (14.2)
Study participant born abroad	224 (13.9)
Self‐reported health problems	
Back pain	369 (22.8)
Depression	218 (13.5)
Gastrointestinal tract symptoms	159 (9.8)
Hypertension	115 (7.1)
Pulmonary disease	88 (5.4)
Rheumatism or other autoimmune disease	76 (4.7)
Osteoarthritis	52 (3.2)
Heart disease	50 (3.1)
Anemia or other blood disease	35 (2.2)
Kidney disease	32 (2.0)
Diabetes	26 (1.6)
Cancer	18 (1.1)
Liver disease	14 (0.9)
Administered vaccine type	
BNT162b2 (Pfizer BioNTech)	1249 (77.3)
mRNA‐1273 (Moderna)	367 (22.7)
Anxiety or depression (pursuant to PHQ‐4)	
PHQ‐4 score, median (IQR)	0 (0–2)
≥ 3 Points in depression	108 (6.7)
≥ 3 Points in anxiety	98 (6.1)
Somatosensory Amplification Scale score, median (IQR)	34 (30–38)
Premedication with analgesics/antipyretics in the last 2 weeks	386 (23.9)

Table 2 shows the distribution of the reported experiences from the first vaccination, as well as expectations and satisfaction regarding the vaccination process at the study site. More than two thirds of all participants experienced no vaccine reactions or only mild vaccine reactions at their first vaccination (Likert values of 0–2). More than 75% of participants expected no risk or only a low risk of severe adverse effects or long‐term adverse effects of the second vaccination (Likert values of 0–3). Overall satisfaction with the vaccination process at the study site was high, with more than 85% of participants indicating high satisfaction (Likert values of 7–10).

**TABLE 2:** Reported experiences, expectations, and satisfaction regarding the vaccination process at baseline.

*Reported experiences (n = 1616)*	
Vaccine reactions experienced at first vaccination (00: no reaction; 10: strongest vaccine reaction)	00: 22.0% 01: 28.8% 02: 18.6% 03: 9.2% 04: 3.7% 05: 4.8% 06: 5.8% 07: 3.9% 08: 1.6% 09: 0.8% 10: 0.8%
*Reported expectations (n = 1616)*	
Expected risk for vaccine reactions (00: no risk; 10: maximum risk)	00: 1.5% 01: 4.0% 02: 8.9% 03: 10.1% 04: 7.7% 05: 15.6% 06: 14.5% 07: 15.5% 08: 12.4% 09: 5.7% 10: 4.1%
Expected risk for hospitalization due to adverse effects of vaccination (00: no risk; 10: maximum risk)	00: 16.8% 01: 29.0% 02: 22.6% 03: 12.9% 04: 4.3% 05: 6.5% 06: 3.7% 07: 1.9% 08: 1.2% 09: 0.6% 10: 0.4%
Expected risk for long‐term adverse effects of vaccination (00: no risk; 10: maximum risk)	00: 19.6% 01: 25.9% 02: 19.1% 03: 10.1% 04: 5.3% 05: 9.9% 06: 4.3% 07: 2.9% 08: 1.6% 09: 0.4% 10: 0.7%
Expected benefit of vaccination (00: no benefit; 10: maximum benefit)	00: 0.7% 01: 0.4% 02: 1.1% 03: 1.4% 04: 0.9% 05: 5.0% 06: 4.7% 07: 11.3% 08: 21.3% 09: 16.0% 10: 37.1%
Expected risk of contraction of COVID‐19 without vaccination (within 12 months) (00: no risk; 10: maximum risk)	00: 3.1% 01: 3.2% 02: 10.8% 03: 12.2% 04: 7.8% 05: 14.6% 06: 7.9% 07: 15.0% 08: 12.3% 09: 4.9% 10: 8.2%
Expected risk for hospitalization due to COVID‐19 without vaccination (within 12 months) (00: no risk; 10: maximum risk)	00: 5.0% 01: 10.8% 02: 15.3% 03: 15.0% 04: 9.8% 05: 12.3% 06: 9.7% 07: 8.6% 08: 7.1% 09: 3.2% 10: 3.2%
Satisfaction with the organization of the vaccination (*n* = 1616) (00: not satisfied; 10: very satisfied)	00: 0.0% 00: 0.1% 02: 0.3% 03: 0.6% 04: 1.0% 05: 2.0% 06: 2.2% 07: 6.9% 08: 12.9% 09: 18.3% 10: 55.8%

### 3.3 Amount of Self‐Medication

**FIGURE 2:**
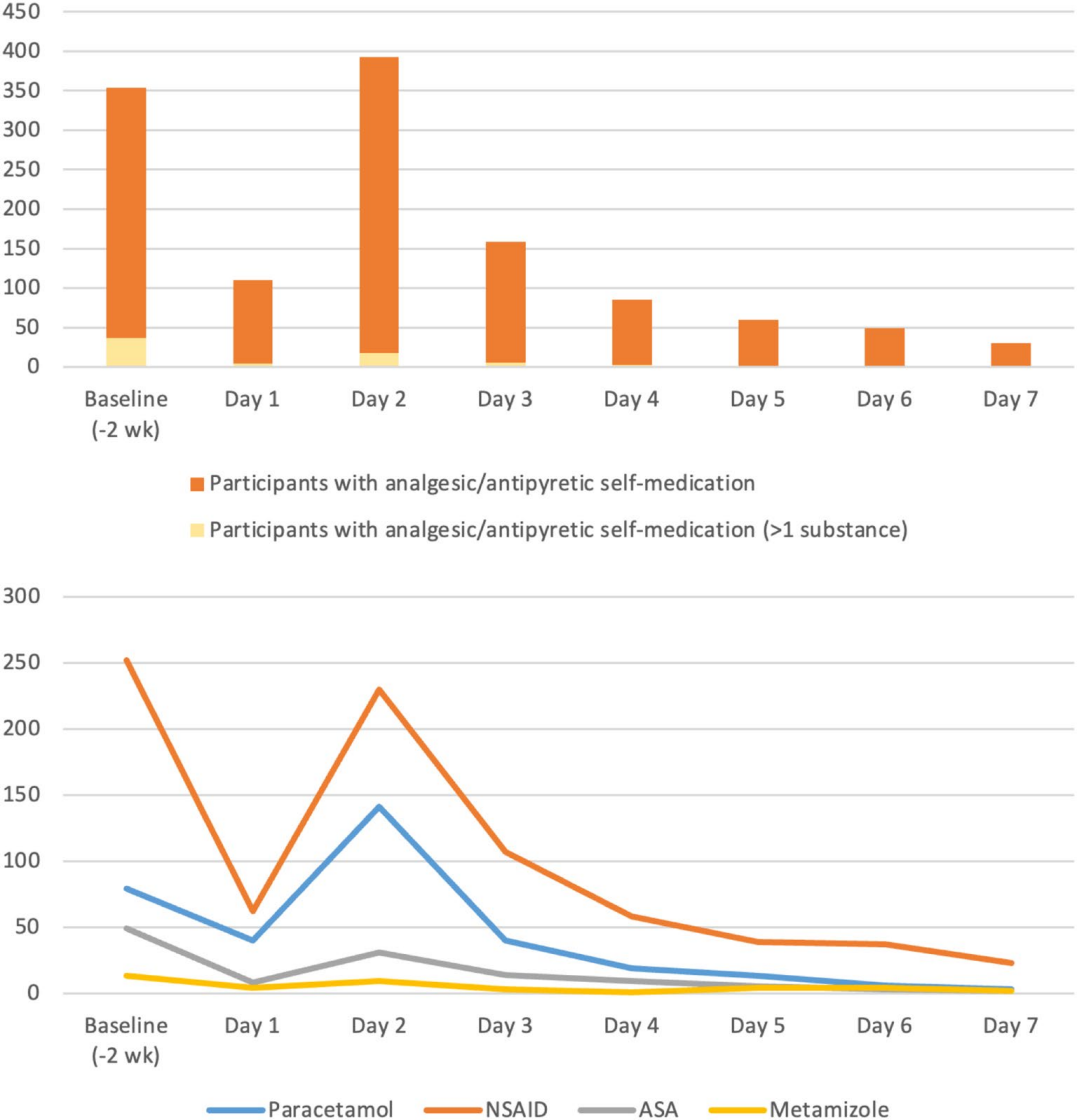
Number of patients practicing self‐medication after vaccination and used substances.

A total number of 536 (33.2%) participants reported the use of self‐medication with analgesics/antipyretics to treat vaccine reactions. Used substances were NSAIDs (*n* = 345; 21.3%), paracetamol (*n* = 186; 11.5%), acetylsalicylic acid (*n* = 48; 3.0%), and metamizole (*n* = 18; 1.1%). Figure 2 depicts the number of participants with usage of analgesics/antipyretics during the study period together with the distribution of administered substances. On the day of vaccination at 9:00 p.m. (Day 1), 110 (6.8%) participants applied self‐medication to mitigate vaccine reactions. On the first day after vaccination at 6:00 p.m. (Day 2), we noted a maximum number of 393 (24.3%) participants with reported self‐medication. In the following days the amount of self‐medication declined continuously to a number of 30 (1.9%) on Day 7. The combined use of two substances was highest on Day 2 with 4.8%. Notably, 354 (21.9%) participants reported an intake of analgesics/antipyretics in the last 2 weeks before vaccination (baseline). NSAIDs were the most common substances in the study period as well as in the baseline period, followed by paracetamol and acetylsalicylic acid. The amount of intake of metamizole (which is available in Germany by prescription only) was lowest. All substances followed the same trend with the highest intake on the first day after vaccination (Day 2) and a continuous decline for the following 5 days (Day 3–7).

### 3.4 Factors of Self‐Medication

We subsequently calculated the number of days under self‐medication with any analgesic/antipyretic and found that *n* = 1080 (66.8%) did not report any self‐medication, whereas *n* = 315 (19.5%) practiced self‐medication for 1 day, *n* = 142 (8.8%) for 2 days, *n* = 42 (2.6%) for 3 days, and *n* = 29 (1.8%) for 4 days. Only *n* = 8 (0.5%) participants utilized analgesics/antipyretics for more than 4 days.

As depicted in Table 3, we conducted a series of bivariate linear regression analyses to evaluate for an unadjusted association of each demographic or psychological factor with the number of days under self‐medication. The strongest association was noted in participants with a baseline intake of any analgesic/antipyretic in the last 2 weeks before vaccination (*B* = 0.651; 95% CI: 0.546; 0.757) followed by female gender (*B* = 0.284; 95% CI: 0.191; 0.377), the occurrence of vaccine reactions after the first vaccination (*B* = 0.21; 95% CI: 0.15; 0.27), and the administration of the mRNA‐1273 vaccine instead of BNT162b2 (*B* = 0.334; 95% CI: 0.223; 0.445). From the list of 12 common health problems, five conditions showed a significant association with the days under self‐medication: gastrointestinal tract symptoms (*B* = 0.334; 95% CI: 0.177; 0.491), back pain (*B* = 0.290; 95% CI: 0.179; 0.401), depression (*B* = 0.299; 95% CI: 0.163; 0.436), pulmonary disease (*B* = 0.322; 95% CI: 0.115; 0.528), and heart disease (*B* = 0.28; 95% CI: 0.009; 0.552). Three items regarding expectations due to the vaccination showed an association with the number of days under self‐medication (per 3‐point difference on a 10‐item Likert scale): the self‐perceived risk for vaccine reactions (*B* = 0.162; 95% CI: 0.105; 0.219), the self‐perceived risk for hospitalization due to the vaccination (*B* = 0.132; 95% CI: 0.063; 0.204) and the risk for long‐term adverse effects (*B* = 0.072; 95% CI: 0.006; 0.135). Notably, the satisfaction with the organization of the process at the vaccination center showed a negative association with the days under self‐medication (*B* = −0.186; 95% CI: −0.282; −0.09).

**TABLE 3:** Unadjusted and adjusted associations (multivariate linear regression with stepwise forward selection) for “days under self‐medication” (*n* = 1616).

Variables	Unadjusted association		Adjusted association	
	*B* (95% CI)	*p*	*B* (95% CI)	*p*
Age (per 10 years)	−0.01 (−0.05;0.30)	0.510		
Gender				
Male	1 [Reference]	NA	1 [Reference]	NA
Female	0.284 (0.191; 0.377)	< 0.001	0.159 (0.07; 0.249)	< 0.001
Living arrangements				
Living with others	1 [Reference]	NA		
Living alone	−0.059 (−0.164; 0.047)	0.276		
Education level (pursuant to CASMIN3)				
Inadequately completed, general elementary or basic vocational	1 [Reference]	NA		
Secondary school certificate or A‐level equivalent	0.036 (−0.195;0.266)	0.762		
Higher or lower tertiary education	−0.071 (−0.298;0.156)	0.539		
Migration status				
Participant and both parents born in Germany	1 [Reference]	NA		
Participant born in Germany and at least 1 parent born abroad	−0.052 (−0.188;0.085)	0.459		
Participant born abroad	0.065 (−0.113;0.242)	0.474		
Anxiety or depression (per 2‐point difference in PHQ‐4 score)	0.090 (0.046; 0.136)	< 0.001		
Somatosensory Amplification Scale (per 5‐point difference)	−0.034 (−0.05; −0.018)	< 0.001		
Premedication				
No premedication at baseline (2 weeks prior vaccination)	1 [Reference]	NA	1 [Reference]	NA
Premedication with any analgesic/antipyretic at baseline (2 weeks prior vaccination)	0.651 (0.546;0.757)	< 0.001	0.546 (0.441; 0.651)	< 0.001
Administered vaccine type				
Vaccine type BNT162b2	1 [Reference]	NA	1 [Reference]	NA
Vaccine type mRNA‐1273	0.334 (0.223; 0.445)	< 0.001	0.293 (0.188; 0.397)	< 0.001
Self‐reported health problems				
Gastrointestinal tract symptoms	0.334 (0.177; 0.491)	< 0.001		
Back pain	0.290 (0.179; 0.401)	< 0.001	0.177 (0.071; 0.284)	0.001
Depression	0.299 (0.163; 0.436)	< 0.001	0.150 (0.019; 0.281)	0.024
Pulmonary disease	0.322 (0.115; 0.528)	0.002		
Hypertension	0.037 (−0.146; 0.220)	0.692		
Rheumatism or other autoimmune disease	0.046 (−0.176; 0.268)	0.685		
Osteoarthritis	0.169 (−0.098; 0.435)	0.214		
Heart disease	0.280 (0.009; 0.552)	0.043		
Anemia or other blood disease	0.257 (−0.065; 0.580)	0.118		
Kidney disease	0.078 (−0.259; 0.416)	0.649		
Diabetes	0.264 (−0.110; 0.637)	0.166		
Cancer	0.007 (−0.441; 0.455)	0.974		
Liver disease	0.095 (−0.412; 0.603)	0.712		
Reported experiences^a^				
Vaccine reactions experienced at first vaccination (per 3‐point difference)	0.21 (0.15; 0.27)	< 0.001	0.117 (0.054; 0.18)	< 0.001
Reported expectations^a^				
Expected risk for vaccine reactions (per 3‐point difference)	0.162 (0.105; 0.219)	< 0.001	0.063 (0.003; 0.123)	0.040
Expected risk for hospitalization due to adverse effects of vaccination (per 3‐point difference)	0.132 (0.063; 0.204)	< 0.001		
Expected risk for long‐term adverse effects of vaccination (per 3‐point difference)	0.072 (0.006; 0.135)	0.029		
Expected benefit of vaccination (per 3‐point difference)	−0.03 (−0.102; 0.042)	0.436		
Expected risk of contraction of COVID‐19 without vaccination (within 12 months) (per 3‐point difference)	−0.015 (−0.066; 0.039)	0.608		
Expected risk for hospitalization due to COVID‐19 without vaccination (within 12 months) (per 3‐point difference)	0.009 (−0.045; 0.063)	0.711		
Satisfaction with the organization of the vaccination^a^	−0.186 (−0.282; −0.09)	< 0.001	−0.129 (−0.222; −0.039)	0.005

^a^ Questionnaire item with 11‐point Likert scale.

In our multiple linear regression model with adjusted associations between variables and the number of days under self‐medication, baseline intake of analgesics/antipyretics (*B* = 0.546 95% CI: 0.441; 0.651) showed the strongest positive association followed by the administration of the vaccine type mRNA‐1273 (*B* = 0.293; 95% CI: 0.188; 0.397) and female gender (*B* = 0.159; 95% CI: 0.07; 0.249). The magnitude of experienced vaccine reactions at the participant's first vaccination (*B* = 0.117; 95% CI: 0.054; 0.18) as well as the magnitude of expectations regarding the vaccine reactions of the second COVID‐19 vaccine shot (*B* = 0.063; 95% CI: 0.003; 0.123) showed a positive association with self‐medication as well. Regarding the questionnaire's list of common health problems, we observed a positive association with self‐medication for a history of back pain (*B* = 0.177; 95% CI: 0.071; 0.284) and depression (*B* = 0.15; 95% CI: 0.019; 0.281). The self‐reported overall satisfaction with the organizational process showed an association with less self‐medication (*B* = −0.129; 95% CI: −0.222; −0.039).

## 4 Discussion

In agreement with our hypothesis, we found various factors that showed an association with a higher rate of self‐medication. A positive association was observed for a reported baseline premedication of analgesics/antipyretics, the magnitude of pre‐experienced vaccine reactions at first vaccination, and the magnitude of negative expectations of vaccine reactions. These associations seem plausible as participants who already experienced vaccine reactions at their first vaccination will supposedly tend to mitigate expected reactions with drugs they are used to. In addition, we observed more self‐medication with the usage of the mRNA‐1273 vaccine type. The mRNA‐1273 vaccine showed a higher reactogenicity in many studies [7, 8]; therefore, more self‐medication due to this vaccine type seems plausible as well. Therefore, we can assume that the expectation as well as the perception of vaccine reactions influenced self‐medication behavior.

The three main mechanisms of Nocebo effects are learning, expectations, and misattribution [27]. We found that all three mechanisms were present in our sample. First, the association of pre‐experiences with self‐medication indicates a learning effect from the first vaccination. Second, the participants' expectations regarding future vaccine reactions were also associated with an intake of analgesics/antipyretics. Third, the positive association between baseline use of analgesics/antipyretics and back pain, as well as psychological complaints like depression, could indicate a misattribution of symptoms from other conditions. Such misattribution may lead to increased use of drugs to which participants were already accustomed.

Back pain and depression comprised more than one third of all reported chronic health problems. This may have contributed to the observed significant association of these conditions with self‐medication in our sample. However, we assume that participants used the term ‘depression’ to refer to a variety of psychological complaints, since our questionnaire included pre‐defined categories of health problems and participants self‐administered the questions using their smartphones. An association of chronic diseases that demand analgesics/antipyretics with self‐medication is reported from various studies [11, 34]. Furthermore, various mental complaints and diseases showed an association with self‐medication [34, 35].

Female participants were more likely to self‐medicate in our sample which is in line with females being more likely to self‐medicate across different countries and age groups [35, 36, 37, 38]. Notably, male participants reported less vaccine reactions of COVID‐19 vaccination in our previously published analysis and other studies [28, 39].

A factor of less self‐medication in our sample was the magnitude of overall satisfaction with the organization at the vaccination center. While this finding aligns with research showing that positive healthcare experiences influence vaccination attitudes and health behavior [40, 41, 42] our satisfaction item captured a broad assessment of procedural aspects rather than specific components such as communication or waiting times.

The observed self‐medication rate of 33.5% in our sample is in line with a recent study of self‐medication during the COVID‐19 pandemic that reported a prevalence for self‐medication of 34.3% in the European union [42]. The distribution of used substances in our cohort is lower than in a recent German study that reported a much higher use of paracetamol, acetylsalicylic acid, and metamizole [43]. The lower rates in our sample may reflect the context of post‐vaccination symptom management, as opposed to broader self‐medication practices for various complaints. However, our findings confirm NSAIDs and paracetamol as the most commonly used agents for symptom management [44].

### 4.1 Strengths and Limitations

A strength of our study is its prospective longitudinal design, which enabled daily assessment of self‐medication over seven consecutive days in a real‐world setting, thereby reducing recall bias. The large sample size and the inclusion of both clinical (e.g., chronic conditions, vaccine type) and psychosocial variables (e.g., prior experiences, expectations, satisfaction) allowed for an analysis of various factors with potential influence on self‐medication behavior, including the role of nocebo effects.

However, several limitations should be mentioned. The response rate of 20.8% was relatively low and the final sample size is smaller than in our previous analysis [28] due to further exclusions for missing data on analgesic/antipyretic usage. Furthermore, a nonresponder analysis was not possible, as excluded participants predominantly lacked the required baseline data. We cannot completely exclude that some reported medication use was for pre‐existing conditions rather than vaccine‐related symptoms, although our observed associations with vaccine‐specific factors support our assumptions.

The self‐reported nature of the data introduces the possibility of reporting bias due to social desirability. The reliance on participants' subjective interpretation of the questionnaire items may have led to imprecise classification of health conditions and experiences. In addition, the high educational status of our sample as well as the younger age does not supposedly represent the general population in Germany. Our sample was maybe more willing than average to use digital tools, which could be a source of selection bias. Finally, although we adjusted for various factors, residual confounding cannot be entirely ruled out.

### 4.2 Implications for Clinical Practice

This study provides insights into demographic, clinical, and psychosocial factors influencing self‐medication behavior. The knowledge of such factors could aid consultations regarding vaccinations and enable physicians to assess the likelihood of self‐medication for their patients by establishing specific screening questions. In case of relevant comorbidities, physicians could concentrate on patients with a higher likelihood of self‐medication and inform them about an adequate intake regimen to ensure their safety. Our results highlight the importance of considering patients' prior experiences and—if possible—positively shaping their expectations to increase satisfaction during consultations that address vaccinations. Conversely, for individuals less likely to self‐medicate, encouraging the appropriate use of analgesics/antipyretics to manage vaccine reactions may help improve overall vaccine acceptance.

## 5 Conclusions

Factors influencing self‐medication behavior with analgesics/antipyretics after vaccination included not only demographic factors such as gender and clinical factors such as vaccine type, premedication, and comorbidities, but also psychosocial factors such as prior experiences, expectations, and satisfaction. Knowledge of these factors may improve consultations between healthcare providers and patients.

## Author Contributions

M.B. and J.H.O.‐A. had full access to all the data in the study and take responsibility for the integrity of the data and the accuracy of the data analysis. J.H.O.‐A., M.B., I.S., Y.N., C.B., and M.S. initiated the study design and developed the survey. M.B., J.H.O.‐A., and M.S. piloted the survey at the outpatient clinic of the Department of General Practice and Primary Care at the University Medical Center Hamburg‐Eppendorf, and M.B., J.H.O.‐A., I.S., Y.N., C.B., and M.S. conducted the study. M.B., J.H.O.‐A., I.S., and M.B. analyzed the data. M.B., J.H.O.‐A., I.S., Y.N., C.B., and M.S. interpreted the results. J.H.O.‐A. and M.B. drafted the manuscript. J.H.O.‐A. is responsible for the overall content. All authors made refinements and approved the final manuscript.

## Funding

This study was supported by grant CRC 289 Treatment Expectation, Project Number 422744262, from the DFG and the Open Access Publication Fund of Universitätsklinikum Hamburg‐Eppendorf.

## Ethics Statement

The study was approved by the local Psychological Ethics Committee at the Center for Psychosocial Medicine of the University Medical Center Hamburg‐Eppendorf on April 28, 2021 (LPEK‐0312).

## Conflicts of Interest

The authors declare no conflicts of interest.

## Supporting information


**Table S1:** Linear regression with all independent variables for collinearity testing for “days under self‐medication.”

**Table S2:** Logistic regression with all independent variables included for “under self‐medication” versus “no self‐medication” (sensitivity analysis).

**Table S3:** Baseline Questionnaire—English Version, translated from German.

## Data Availability

The consent statement did not specify that data would be published, and for this reason we are unable to deposit the data set in a public repository. However, de‐identified data are available upon reasonable request from the corresponding author.
