# Supplementary material for: Factors Associated With Self‐Medication to Mitigate Vaccine Reactions After COVID‐19 Vaccination: A Prospective Cohort Study
Source: Pharmacoepidemiol Drug Saf. 2026 Apr 10;35(4):e70372. doi: 10.1002/pds.70372 (PMC13067796; doi:10.1002/pds.70372)
Supplement: Supplementary file 1 — Table S1: Linear regression with all independent variables for collinearity testing for “days under self‐medication.” [file PDS-35-e70372-s003.docx]

Supplementary Table 1

Linear regression with all independent variables for collinearity testing for ´days under self-medication´

|  | Unstandardized Coefficients | Standardized Coefficients | Sig. | 95% CI for B | | Collinearity Statistics | |
| --- | --- | --- | --- | --- | --- | --- | --- |
|  | B | ß |  | Lower Bound | Upper Bound | Tolerance | VIF |
| (Constant) | 0.356 |  | 0.177 | -0.161 | 0.873 |  |  |
| Age | 0.001 | 0.010 | 0.704 | -0.003 | 0.005 | 0.796 | 1.256 |
| Female gender | 0.174 | 0.090 | 0.000 | 0.079 | 0.270 | 0.833 | 1.200 |
| Living alone | -0.071 | -0.033 | 0.163 | -0.172 | 0.029 | 0.951 | 1.052 |
| CASMIN, level 2 | 0.031 | 0.016 | 0.778 | -0.187 | 0.250 | 0.166 | 6.035 |
| CASMIN, level 3 | -0.058 | -0.030 | 0.604 | -0.277 | 0.161 | 0.161 | 6.228 |
| Participant born in Germany and at least 1 parent born abroad | -0.049 | -0.023 | 0.462 | -0.178 | 0.081 | 0.562 | 1.780 |
| Participant born abroad | 0.057 | 0.020 | 0.510 | -0.112 | 0.225 | 0.560 | 1.787 |
| PHQ4-Score | -0.005 | -0.010 | 0.703 | -0.029 | 0.020 | 0.730 | 1.370 |
| Somatosensory Amplification Scale | 0.002 | 0.012 | 0.644 | -0.007 | 0.011 | 0.766 | 1.306 |
| Premedication at baseline | 0.552 | 0.244 | 0.000 | 0.445 | 0.659 | 0.918 | 1.089 |
| Vaccine reactions experienced at first vaccination | 0.037 | 0.087 | 0.001 | 0.015 | 0.059 | 0.767 | 1.303 |
| Heart disease | 0.199 | 0.036 | 0.150 | -0.072 | 0.469 | 0.867 | 1.154 |
| Hypertension | -0.041 | -0.011 | 0.665 | -0.227 | 0.145 | 0.834 | 1.199 |
| Pulmonary disease | 0.148 | 0.035 | 0.145 | -0.051 | 0.347 | 0.930 | 1.075 |
| Diabetes | -0.037 | -0.005 | 0.846 | -0.408 | 0.335 | 0.870 | 1.149 |
| Gastrointestinal tract symptoms | 0.145 | 0.045 | 0.068 | -0.011 | 0.301 | 0.880 | 1.136 |
| Kidney disease | -0.182 | -0.026 | 0.279 | -0.513 | 0.148 | 0.898 | 1.113 |
| Liver disease | 0.070 | 0.007 | 0.775 | -0.412 | 0.553 | 0.952 | 1.051 |
| Anemia or other blood disease | 0.118 | 0.018 | 0.454 | -0.191 | 0.426 | 0.944 | 1.060 |
| Cancer | -0.037 | -0.004 | 0.870 | -0.475 | 0.402 | 0.897 | 1.115 |
| Depression | 0.147 | 0.052 | 0.048 | 0.001 | 0.293 | 0.766 | 1.305 |
| Osteoarthritis | -0.012 | -0.002 | 0.931 | -0.274 | 0.251 | 0.886 | 1.128 |
| Back pain | 0.144 | 0.063 | 0.013 | 0.030 | 0.257 | 0.839 | 1.192 |
| Rheumatism or other autoimmune disease | -0.121 | -0.027 | 0.270 | -0.336 | 0.094 | 0.917 | 1.090 |
| Expected risk for vaccine reactions | 0.022 | 0.055 | 0.046 | 0.000 | 0.043 | 0.699 | 1.430 |
| Expected risk for hospitalization due to adverse effects of vaccination | 0.024 | 0.050 | 0.119 | -0.006 | 0.055 | 0.525 | 1.904 |
| Expected risk for long-term adverse effects of vaccination | -0.019 | -0.043 | 0.165 | -0.046 | 0.008 | 0.549 | 1.822 |
| Expected benefit of vaccination | 0.009 | 0.018 | 0.508 | -0.017 | 0.035 | 0.712 | 1.404 |
| Expected risk of contraction of COVID-19 without vaccination (within 12 months) | -0.005 | -0.013 | 0.641 | -0.024 | 0.015 | 0.671 | 1.490 |
| Expected risk for hospitalization due to COVID-19 without vaccination (within 12 months) | -0.013 | -0.035 | 0.245 | -0.034 | 0.009 | 0.593 | 1.686 |
| Satisfaction with the organization of the vaccination | -0.046 | -0.070 | 0.005 | -0.079 | -0.014 | 0.861 | 1.162 |
| Vaccine Type mRNA-1273 | 0.292 | 0.127 | <0.001 | 0.185 | 0.398 | 0.955 | 1.047 |
