# Supplementary material for: Factors Associated With Self‐Medication to Mitigate Vaccine Reactions After COVID‐19 Vaccination: A Prospective Cohort Study
Source: Pharmacoepidemiol Drug Saf. 2026 Apr 10;35(4):e70372. doi: 10.1002/pds.70372 (PMC13067796; doi:10.1002/pds.70372)
Supplement: Supplementary file 2 — Table S2: Logistic regression with all independent variables included for “under self‐medication” versus “no self‐medication” (sensitivity analysis). [file PDS-35-e70372-s001.docx]

Supplementary Table 2

Logistic regression with all independent variables included for ´under self-medication´ vs. ´no self-medication´ (sensitivity analysis)

|  | Sig. | OR | 95% C.I. for OR | |
| --- | --- | --- | --- | --- |
|  |  |  | Lower | Upper |
| Age | 0.277 | 0.994 | 0.983 | 1.005 |
| Female gender | 0.000 | 1.608 | 1.259 | 2.053 |
| Living alone | 0.132 | 0.818 | 0.630 | 1.062 |
| CASMIN, level 2 | 0.596 | 1.168 | 0.659 | 2.070 |
| CASMIN, level 3 | 0.961 | 1.015 | 0.571 | 1.802 |
| Participant born in Germany and at least 1 parent born abroad | 0.284 | 0.833 | 0.596 | 1.164 |
| Participant born abroad | 0.118 | 1.408 | 0.917 | 2.162 |
| PHQ4-Score | 0.257 | 0.965 | 0.907 | 1.027 |
| Somatosensory Amplification Scale | 0.968 | 1.000 | 0.979 | 1.022 |
| Premedication at baseline | <0.001 | 3.073 | 2.379 | 3.971 |
| Vaccine reactions experienced at first vaccination | 0.018 | 1.067 | 1.011 | 1.126 |
| Heart disease | 0.347 | 1.387 | 0.702 | 2.741 |
| Hypertension | 0.708 | 0.913 | 0.566 | 1.471 |
| Pulmonary disease | 0.977 | 1.007 | 0.611 | 1.661 |
| Diabetes | 0.719 | 1.182 | 0.475 | 2.940 |
| Gastrointestinal tract symptoms | 0.330 | 1.209 | 0.825 | 1.771 |
| Kidney disease | 0.394 | 0.691 | 0.296 | 1.615 |
| Liver disease | 0.973 | 0.979 | 0.279 | 3.435 |
| Anemia or other blood disease | 0.354 | 1.430 | 0.671 | 3.048 |
| Cancer | 0.519 | 0.675 | 0.204 | 2.233 |
| Depression | 0.173 | 1.285 | 0.896 | 1.845 |
| Osteoarthritis | 0.565 | 0.818 | 0.414 | 1.619 |
| Back pain | 0.072 | 1.295 | 0.977 | 1.716 |
| Rheumatism or other autoimmune disease | 0.442 | 0.804 | 0.462 | 1.401 |
| Expected risk for vaccine reactions | 0.005 | 1.082 | 1.024 | 1.143 |
| Expected risk for hospitalization due to adverse effects of vaccination | 0.702 | 0.985 | 0.912 | 1.064 |
| Expected risk for long-term adverse effects of vaccination | 0.471 | 0.975 | 0.911 | 1.044 |
| Expected benefit of vaccination | 0.832 | 0.993 | 0.929 | 1.061 |
| Expected risk of contraction of COVID-19 without vaccination (within 12 months) | 0.636 | 0.988 | 0.939 | 1.039 |
| Expected risk for hospitalization due to COVID-19 without vaccination (within 12 months) | 0.155 | 0.960 | 0.908 | 1.015 |
| Satisfaction with the organization of the vaccination | 0.405 | 0.966 | 0.892 | 1.047 |
| Vaccine Type mRNA-1273 | <0.001 | 2.202 | 1.699 | 2.855 |
| Constant | 0.072 | 0.297 |  |  |
