# Supplementary material for: Factors Associated With Self‐Medication to Mitigate Vaccine Reactions After COVID‐19 Vaccination: A Prospective Cohort Study
Source: Pharmacoepidemiol Drug Saf. 2026 Apr 10;35(4):e70372. doi: 10.1002/pds.70372 (PMC13067796; doi:10.1002/pds.70372)
Supplement: Supplementary file 3 — Table S3: Baseline Questionnaire—English Version, translated from German. [file PDS-35-e70372-s002.docx]

**Supplementary Table 3**

**Baseline Questionnaire — English Version, Translated from German**

| **Please indicate which fever-reducing or pain-relieving medication you took during the past 14 days (multiple answers possible):** |
| --- |
| - No medication - Paracetamol - Ibuprofen / Naproxen / Diclofenac - Metamizole (Novalgin) - ASA / Aspirin |

**Mental Well-being (Past two Weeks)**

| **How often during the past two weeks have you been bothered by the following problems?** | **Not at all** | **On several days** | **On more than halt the days** | **Nearly every day** |
| --- | --- | --- | --- | --- |
| **PHQ1** **Little interest or pleasure in doing things** |  |  |  |  |
| **PHQ2** **Feeling down, depressed, or hopeless** |  |  |  |  |
| **PHQ3** **Nervousness, anxiety, or tension** |  |  |  |  |
| **PHQ4** **Not being able to stop or control worrying** |  |  |  |  |

| **Which vaccine did you receive today?** |
| --- |
| Biontec/Pfizer |
| AstraZeneca |
| Moderna |
| Johnson & Johnson |

| **How do you estimate your risk of experiencing vaccine reactions (e.g., fever, headache, body aches)?** |
| --- |

| **no risk** | **0** | **1** | **2** | **3** | **4** | **5** | **6** | **7** | **8** | **9** | **10** | **maximum risk** |
| --- | --- | --- | --- | --- | --- | --- | --- | --- | --- | --- | --- | --- |

| **How do you estimate your risk of experiencing serious adverse effects from the vaccination (requiring hospitalization)?** |
| --- |

| **no risk** | **0** | **1** | **2** | **3** | **4** | **5** | **6** | **7** | **8** | **9** | **10** | **maximum risk** |
| --- | --- | --- | --- | --- | --- | --- | --- | --- | --- | --- | --- | --- |

| **How do you estimate your personal risk of suffering undesirable long-term averse effects from the vaccination?** |
| --- |

| **no risk** | **0** | **1** | **2** | **3** | **4** | **5** | **6** | **7** | **8** | **9** | **10** | **maximum risk** |
| --- | --- | --- | --- | --- | --- | --- | --- | --- | --- | --- | --- | --- |

| **How high do you estimate the personal benefit of your COVID-19 vaccination?** |
| --- |

| **no benefit** | **0** | **1** | **2** | **3** | **4** | **5** | **6** | **7** | **8** | **9** | **10** | **maximum benefit** |
| --- | --- | --- | --- | --- | --- | --- | --- | --- | --- | --- | --- | --- |

| **How do you estimate your personal risk of contracting COVID-19 within the next twelve months without vaccination?** |
| --- |

| **no risk** | **0** | **1** | **2** | **3** | **4** | **5** | **6** | **7** | **8** | **9** | **10** | **maximum risk** |
| --- | --- | --- | --- | --- | --- | --- | --- | --- | --- | --- | --- | --- |

| **How do you estimate your risk of contracting COVID-19 and experiencing a severe course (hospitalization) within the next twelve months without vaccination?** |
| --- |

| **no risk** | **0** | **1** | **2** | **3** | **4** | **5** | **6** | **7** | **8** | **9** | **10** | **maximum risk** |
| --- | --- | --- | --- | --- | --- | --- | --- | --- | --- | --- | --- | --- |

| **Is this your second COVID-19 vaccination?** |
| --- |
| No |
| Yes |

| **How severe were the vaccine reactions you experienced after your first COVID-19 vaccination?** |
| --- |

| **no complaints** | **0** | **1** | **2** | **3** | **4** | **5** | **6** | **7** | **8** | **9** | **10** | **maximum discomfort** |
| --- | --- | --- | --- | --- | --- | --- | --- | --- | --- | --- | --- | --- |

| **How satisfied are you with the entire process of your vaccination appointment today?** |
| --- |

| **Not satisfied** | **0** | **1** | **2** | **3** | **4** | **5** | **6** | **7** | **8** | **9** | **10** | **Very satisfied** |
| --- | --- | --- | --- | --- | --- | --- | --- | --- | --- | --- | --- | --- |

**The following questionnaire contains a series of statements about bodily sensations that you can use to describe yourself. Please read each statement and indicate how well it generally applies to you.**

|  | **Comp-letely true** | **Mostly true** | **Somewhat true** | **Mostly not true** | **Not true** |
| --- | --- | --- | --- | --- | --- |
| **SSA01** I cannot tolerate smoke or air pollution |  |  |  |  |  |
| **SSA02** I am often aware of what is going on in my body |  |  |  |  |  |
| **SSA03** Bruises remain visible on me for a long time |  |  |  |  |  |
| **SSA04** I sometimes feel blood flowing through my body |  |  |  |  |  |
| **SSA05** Sudden loud noises bother me greatly |  |  |  |  |  |
| **SSA06** I can often hear my heartbeat in my ears |  |  |  |  |  |
| **SSA07** I feel extremely uncomfortable when too cold or too hot |  |  |  |  |  |
| **SSA08** I quickly notice hunger-related stomach sensations |  |  |  |  |  |
| **SSA09** Minor things like insect bites bother me |  |  |  |  |  |
| **SSA10** I have difficulty tolerating pain |  |  |  |  |  |

**Next, we would like to ask you a few questions about yourself.**

| **In which month and year were you born?** |
| --- |
| ⎣ ⎦ ⎣ ⎦. ⎣ ⎦ ⎣ ⎦ ⎣ ⎦ ⎣ ⎦ |

| **What is your gender?** |
| --- |
| female |
| male |
| diverse |

| **Do you live alone?** |
| --- |
| no |
| yes |

| **With whom else do you live in the same household? (Multiple answers possible)** |
| --- |
| with my spouse  with my child(ren)  with my own parents or my partner's parents  with other family members  with other people (e.g., acquaintances, roommates, etc.) |
| **What is your highest general education qualification?** |
| No school leaving certificate  Graduation lower secondary school,  elementary school, or 8-year primary school (former GDR before 1965)  Intermediate school leaving certificate, or graduation from  intermediate secondary school or Polytechnische Oberschule (POS)  University of Applied Sciences entrance qualification  Abitur (university entrance qualification)  Other school leaving certificate, namely: |

| **What is your highest professional qualification?** |
| --- |
| Vocational training (apprenticeship with vocational school)  Vocational school-based training (vocational college, commercial school)  Technical college, master craftsman school, technical school, vocational academy or specialized academy  University of Applied Sciences (Bachelor's, Master's, state examination, diploma; incl. universities of applied sciences)  University (Bachelor's, Master's, Magister, state examination, diploma)  Other professional qualification, namely: |

| **In which country were you born?** *Please take into account the national borders at that time.* |
| --- |
| in Germany |
| In another country, namely ⎣ ⎦ |

| **In which country were your parents born?** *Please take into account the national borders at that time.* |
| --- |
| **Mother**  in Germany  In another country, namely ⎣ ⎦ |
| **Father**  in Germany  In another country, namely ⎣ ⎦ |

**The following list contains common health problems. Please indicate whether you are under medical treatment for each of the listed conditions.**

|  | **no** | **yes** |
| --- | --- | --- |
|  |  |  |
| **Heart disease** |  |  |
|  |  |  |
| **High blood pressure** |  |  |
|  |  |  |
| **Lung disease** |  |  |
|  |  |  |
| **Diabetes** |  |  |
|  |  |  |
| **Gastrointestinal disorders** |  |  |
|  |  |  |
| **Kidney disease** |  |  |
|  |  |  |
| **Liver disease** |  |  |
|  |  |  |
| **Anemia or blood disorders** |  |  |
|  |  |  |
| **Cancer** |  |  |
|  |  |  |
| **Depression** |  |  |
|  |  |  |
| **Osteoarthritis** |  |  |
|  |  |  |
| **Back pain** |  |  |
|  |  |  |
| **Rheumatic/autoimmune diseases** |  |  |
|  |  |  |

**Thank you very much for taking the time to complete this questionnaire!**

Please hand in this questionnaire together with the signed consent form at the reception of the vaccination center.
